# Parental Mental Health, Feeding Practices, and Sociodemographic Factors as Determinants of Childhood Obesity in Greece

**DOI:** 10.3390/nu18020364

**Published:** 2026-01-22

**Authors:** Vlasia Stymfaliadi, Yannis Manios, Odysseas Androutsos, Maria Michou, Eleni Angelopoulou, Xanthi Tigani, Panagiotis Pipelias, Styliani Katsouli, Christina Kanaka-Gantenbein

**Affiliations:** 1 Postgraduate Course “Science of Stress and Health Promotion”, Medical School, National and Kapodistrian University of Athens (NKUA), 11527 Athens, Greece; vstymf@gmail.com (V.S.); mariamixou@hotmail.com (M.M.); xanthitig@med.uoa.gr (X.T.); panospt@med.uoa.gr (P.P.); stellakatsouli13@gmail.com (S.K.); 2 Department of Nutrition and Dietetics, Harokopio University of Athens, 17676 Athens, Greece; manios@hua.gr; 3 Laboratory of Nutrition & Clinical Dietetics, Department of Dietetics and Nutrition, School of Physical Education, Sport and Dietetics, University of Thessaly, 42132 Trikala, Greece; oandroutsos@uth.gr; 4 First Department of Pediatrics, Medical School, National and Kapodistrian University of Athens, “Aghia Sofia” Children’s Hospital, 11527 Athens, Greece; elen.angel@yahoo.gr

**Keywords:** childhood obesity, parental stress, feeding practices, body mass index (BMI), psychosocial determinants, salivary cortisol, World Health Organization (WHO), Greece

## Abstract

**Background/Objectives:** Childhood obesity remains a major public health issue, particularly in Mediterranean countries such as Greece. Although parental influences on children’s weight have been extensively studied, fewer studies have jointly examined parental mental health, feeding practices, sociodemographic factors, and biological stress markers. This study aimed to investigate associations between psychological status, educational level, feeding behaviors, and children’s Body Mass Index (BMI) in a Greek sample. A pilot assessment of salivary cortisol was included in evaluating its feasibility as an objective biomarker of parental stress. **Subjects and Methods:** A total of 103 parent–child dyads participated in this cross-sectional study. Children’s BMI was classified using World Health Organization (WHO) growth standards. Parental stress, anxiety, and depressive symptoms were assessed using the Perceived Stress Scale-14 (PSS-14) and the Depression Anxiety Stress Scale-21 (DASS-21) questionnaires. Feeding practices were evaluated with the Comprehensive Feeding Practices Questionnaire (CFPQ). Statistical analyses included Pearson correlations, independent samples *t*-tests, one-way ANOVA, Mann–Whitney U, and Kruskal–Wallis tests. A subsample provided saliva samples for cortisol analysis to assess feasibility and explore the potential associations with parental stress indicators. **Results:** Parental BMI showed a strong positive association with child BMI (*p* = 0.002). Higher parental anxiety (*p* = 0.002) and depression (*p* = 0.009) were also associated with increased child BMI. Restrictive (*p* < 0.001) and emotion-driven (*p* < 0.001) feeding practices were associated with higher child BMI, whereas monitoring (*p* = 0.013) and health-promoting feeding practices (*p* = 0.001) appeared protective. Lower parental education was related to a higher BMI in both parents (*p* = 0.001) and children (*p* = 0.002) and to more frequent use of restrictive feeding strategies (*p* = 0.001). WHO charts identified a greater proportion of children as overweight or obese compared with the Centers for Disease Control and Prevention (CDC) criteria. The analysis showed statistically significant differences between the two classification systems (χ^2^ (4) = 159.704, *p* < 0.001), indicating that BMI categorization varies considerably depending on the reference system used. No significant associations were observed with residential environment or salivary cortisol, likely due to the limited size of the pilot biomarker subsample. **Conclusions:** The findings highlight the combined effect of parental mental health status, educational level, and feeding practices on child BMI within the Greek context. The preliminary inclusion of a biological stress marker provides added value to the existing research in this area. These results underscore the importance of prevention strategies that promote parental psychological wellbeing and responsive feeding practices while addressing socioeconomic disparities to reduce the childhood obesity risk.

## 1. Introduction

Childhood obesity has emerged as one of the most urgent global public health challenges, with long-term consequences for children’s physical, psychological, and metabolic health [1]. According to the World Health Organization (WHO), the worldwide prevalence of overweight and obesity among children and adolescents has dramatically risen in recent years, affecting millions of youths across low-, middle-, and high-income countries [2,3,4]. Europe has experienced similar upward trajectories, with Southern European countries reporting the highest burden [5]. Greece consistently ranks among the countries with the highest prevalence of childhood overweight and obesity, with national surveillance systems indicating persistently elevated rates that render effective, targeted, evidence-based public health approaches of utmost importance.

Children’s eating behaviors and weight evolution are shaped largely within the family environment, where parental roles, household dynamics, and dietary routines exert profound influence [6]. Parental feeding practices—defined as the strategies parents use to manage, guide, or regulate their child’s eating—represent a key component of this environment [7]. These practices are not simply behaviorally driven; they are strongly shaped by parental psychology, stress levels, cultural expectations, and socioeconomic pressures [8,9,10]. Accumulating evidence suggests that parental stress plays a key role in modulating feeding behaviors, often in ways that may compromise children’s ability to self-regulate intake and maintain a healthy weight [11,12,13]. Stress can arise from multiple adversities, including financial strain, professional demands, caregiving burdens, family conflict, and limited social support, all of which may modify the consistency, responsiveness, and emotional status of parents’ feeding interactions with their children [14].

Research has shown that higher levels of parental stress are associated with less structured and less responsive feeding practices, such as increased pressure to eat, inconsistent limit setting, emotional feeding, greater use of food as reward or comfort, and reduced monitoring of unhealthy foods [15,16,17,18]. These non-responsive practices have been linked to disinhibited eating, emotional overeating, and diminished satiety responsiveness in children, thereby contributing to excess weight gain over time [19,20,21]. Conversely, responsive feeding—characterized by supportive guidance, modeling healthy choices, and providing autonomy—has been consistently associated with healthier dietary habits and more optimal weight trajectories during childhood [22,23,24].

Stress is a complex biopsychosocial process involving neuroendocrine, cognitive, emotional, and behavioral pathways [25]. Central to the biological stress response is the activation of the hypothalamic–pituitary–adrenal (HPA) axis, which leads to the release of glucocorticoids, primarily cortisol [26]. Chronic or dysregulated cortisol secretion has been associated with altered appetite control, preferences for energy-dense foods, increased reward-driven eating, abdominal adiposity, reduced physical activity, sleep disturbances, and disruptions in metabolic homeostasis—pathways all implicated in obesity development [27,28,29,30]. Stress-related physiological changes can also affect gastrointestinal function, systemic inflammation, and energy balance, further supporting the link between chronic stress exposure and weight gain in both adults and children [31,32,33].

Within the family context, parental stress has both direct and indirect implications for children’s health. Increased parental stress can amplify children’s own physiological and emotional reactivity, diminish parental emotional availability, reduce positive communication, increase irritability or inconsistency in parenting, and contribute to more conflictual or chaotic home environments [34,35,36,37]. These conditions may limit opportunities for structured family meals, reduce shared physical activity, increase children’s sedentary screen time, and weaken routines that support healthy behaviors [38,39,40]. Parental stress may further aggravate parental mental health, including symptoms of anxiety and depression, which may provoke difficulties in maintaining supportive feeding practices or establishing predictable household routines [38,41,42]. Co-parenting quality further moderates these processes, with supportive and cooperative parenting acting as a buffer against stress-related disruptions in family functioning [43].

Given these complex interactions, parental feeding practices represent a critical mechanism linking stress to children’s dietary behaviors and weight outcomes. The Comprehensive Feeding Practices Questionnaire (CFPQ) and similar validated tools have been widely used to assess the multidimensional aspects of feeding, including positive practices (e.g., monitoring, modeling, healthy-eating guidance) and maladaptive strategies (e.g., pressure to eat, restriction, emotional feeding, food reward) [44,45,46]. Evidence consistently indicates that responsive and autonomy-supportive feeding promotes healthier eating behaviors and sustainable self-regulation of energy intake among children [47,48,49]. In contrast, coercive or emotionally driven practices may undermine children’s satiety cues, promote overeating, and contribute to unhealthy weight gain [50,51,52].

In parallel with these family-level dynamics, epidemiological evidence underscores the growing global and national burden of childhood obesity. Worldwide, the prevalence of overweight and obesity among school-aged children has increased more than eight-fold from 1975 to 2016, with substantial rises across regions regardless of economic development [53,54,55]. Socioeconomic inequalities further exacerbate these trends, as children from disadvantaged households face disproportionate exposure to obesogenic environments, reduced access to healthy foods, and greater psychosocial stress [56,57,58]. In Greece, national surveys such as the World Health Organization (WHO) European Childhood Obesity Surveillance Initiative (COSI) and the Greek Examination of Cohorts (GRECO) Study have consistently reported overweight and obesity rates exceeding 35–40% among primary school children, among the highest in Europe [7,59,60]. These alarming trends reflect the interplay between biological predispositions, obesogenic environments, family behaviors, sociocultural norms, and psychosocial determinants including parental stress and mental health [61,62,63].

In the Greek context, childhood obesity is closely linked to family-centered lifestyles, strong parental involvement in child feeding, and sociocultural norms that emphasize shared meals and caregiving roles. At the same time, prolonged economic strain and social stressors in recent decades may further influence psychological wellbeing and feeding behaviors, highlighting the importance of examining these associations within a national framework.

Despite extensive research linking stress, mental health, and obesity, fewer studies have thoroughly examined how parental psychological functioning interacts with feeding practices, sociodemographic characteristics, and children’s anthropometric outcomes within the same analytical framework [64,65,66]. Even fewer studies have explored these relationships in Mediterranean populations, where traditional dietary habits coexist with rapid lifestyle changes, high obesity prevalence, and unique familial caregiving dynamics [53,67,68]. The integration of biological stress markers, such as salivary cortisol, into family-based pediatric obesity research remains limited, although such biomarkers provide valuable objective insight into the physiological stress pathways that may influence parenting behavior and child health [69,70].

Although childhood obesity has been extensively studied, existing research has predominantly examined parental psychological factors, feeding practices, and sociodemographic characteristics as separate determinants of child weight outcomes. Fewer studies have approached these domains in an integrated manner, particularly within Mediterranean populations, where cultural, familial, and social contexts may shape parental behaviors differently. Moreover, evidence remains limited regarding the concurrent consideration of behavioral and biological indicators of stress in relation to childhood obesity.

The present study addresses these gaps by investigating the associations among parental stress, parental mental health, feeding practices, and children’s weight status in a Greek sample of parent–child dyads. By incorporating both psychosocial assessments and a preliminary biological measure of parental stress (salivary cortisol), this research offers an integrated examination of the behavioral, psychological, and physiological pathways that may contribute to childhood obesity within contemporary Mediterranean contexts [71,72]. Understanding these interconnected determinants is essential for informing family-centered interventions and public health strategies aimed at mitigating the childhood obesity epidemic.

Based on the existing evidence, it was hypothesized that higher levels of parental stress and poorer parental mental health would be associated with less responsive feeding practices and higher child Body Mass Index. In addition, maladaptive feeding practices were expected to be positively associated with children’s weight status. These hypotheses were examined within the context of relevant sociodemographic characteristics.

## 2. Subjects and Methods

### 2.1. Study Design and Ethical Approval

This cross-sectional study was conducted between 2024 and 2025 in community settings in the regions of Attica and Corinthia, as well as at the Pediatric Obesity Clinic of the First Department of Pediatrics, Medical School of the National and Kapodistrian University of Athens (NKUA), at “Aghia Sofia” Children’s Hospital, Athens, Greece. The study adhered to the ethical standards described in the Declaration of Helsinki.

The study protocol was approved by the Scientific Committee of the “Aghia Sofia” Children’s Hospital (initial approval: protocol no. 19998/05.08.2024, approved on 11 October 2024; protocol amendment: protocol no. 13710/05.06.2025, approved on 17 June 2025). Written informed consent was obtained from all participating parents or legal guardians prior to data collection.

### 2.2. Procedure

Parents were invited to participate either in community settings or during visits to the Pediatric Obesity Clinic. After receiving standardized information about the study aims and procedures, written informed consent was obtained from all participating parents or legal guardians. Parents completed paper-based questionnaires, including a sociodemographic form and validated instruments assessing perceived stress, mental health, and feeding practices (approximately 20 min). Children’s anthropometric data were recorded as part of the study assessment. In a pilot subsample, salivary cortisol samples were collected following standardized instructions and analyzed in the affiliated laboratory. All data were coded and handled confidentially in accordance with ethical and data protection principles.

All psychometric instruments used in this study (PSS-14, DASS-21, and CFPQ) were administered in their officially translated and validated Greek versions. Prior to data collection, written permission was obtained from the respective developers and/or copyright holders of each instrument.

### 2.3. Participants

The study included 103 parent–child dyads, consisting of 23 father–child dyads and 80 mother–child dyads, with children aged 2–12 years. Participants were recruited either during clinic visits or through community outreach.

Inclusion Criteria

- Parents of children aged 2–12 years.
- Group A: Children with normal weight (5th–85th percentile) or underweight (<5th percentile).
- Group B: Children with overweight (>85th percentile), obesity (>95th percentile), or severe obesity based on age- and sex-specific BMI percentiles.

Exclusion Criteria

- Secondary causes of obesity (e.g., endocrine disorders such as Cushing syndrome or growth hormone deficiency).
- Genetic syndromes associated with abnormal weight (e.g., Down syndrome, Prader–Willi syndrome, etc.).
- Chronic diseases or severe emotional/behavioral disorders.

Participant Flow

A total of 130 parents were approached; after applying inclusion and exclusion criteria, 103 dyads were included in the final analysis.

Anthropometric Measurements

Child height and weight were obtained either by trained clinical staff at the Pediatric Obesity Clinic or parent-reported for community participants following standardized written instructions.

BMI was calculated as weight (kg)/height (m^2^).

- For ages 2–5 years, BMI-for-age z-scores were computed using the WHO child growth standards (2006) [73] and WHO Anthro software (version 3.2.2) [74].
- For ages 5–12 years, BMI-for-age z-scores were computed using the WHO growth reference (2007) [75] and WHO AnthroPlus software (version 1.0.4) [76].

For cross-method comparability, BMI categories were additionally verified using the CDC Child and Teen BMI Calculator [77].

### 2.4. Measures

Sociodemographic Questionnaire

A structured questionnaire was used to collect information on parental sex, age, education, occupation, BMI, residence, and child characteristics (age, sex, height, weight, health history).

Perceived Stress Scale (PSS-14)

Parental perceived stress was assessed using the Greek-validated version of the Perceived Stress Scale-14 (PSS-14) [78], originally developed by Cohen et al. [79], a 14-item measure evaluating the frequency of stress-related thoughts and feelings on a 0–4 Likert scale, yielding total scores from 0 to 56. Higher total scores indicate higher perceived stress.

Depression Anxiety Stress Scale (DASS-21)

The Greek-validated Depression Anxiety Stress Scale-21 (DASS-21) [80], originally developed by Lovibond et al. [81], was used to assess symptoms of depression, anxiety, and stress. The scale includes 21 items rated on a 0–3 Likert scale and produces three 7-item subscale scores. Separate scores were calculated for each subscale (Depression, Anxiety, and Stress), with higher scores indicating greater symptom severity.

Comprehensive Feeding Practices Questionnaire (CFPQ)

Parent feeding practices were measured using the Greek-validated Comprehensive Feeding Practices Questionnaire (CFPQ) [82], originally developed by Musher-Eizenman et al. [17], consisting of 42 items across six factors:

1. Healthy Eating Guidance (14 items);
2. Emotion Regulation/Food as Reward (6 items);
3. Monitoring (4 items);
4. Child Control (5 items);
5. Pressure (3 items);
6. Restriction (10 items).

Subscale scores were calculated by summing item responses within each factor, with higher scores reflecting greater use of the corresponding feeding practice.

Pilot Salivary Cortisol Assessment

A subsample of 13 parents provided four salivary cortisol samples (awakening, +30–45 min, evening, midnight) collected using Salivette^®^ (Sarstedt, Nümbrecht, Germany) devices. Samples were centrifuged, stored at −20 °C, and analyzed in batch using a validated immunoassay at the Clinical and Translational Endocrinology Laboratory, “Aghia Sofia” Children’s Hospital. This assessment served as a feasibility pilot to explore the biological markers of parental stress.

### 2.5. Statistical Analysis

Quantitative variables are presented as means ± standard deviation or medians (interquartile range), depending on distribution. Categorical variables are presented as frequencies (%). Normality was assessed using the Kolmogorov–Smirnov test. Group comparisons were conducted using independent sample *t*-tests and one-way ANOVA for normally distributed variables, and Mann–Whitney U and Kruskal–Wallis tests for non-normally distributed variables.

Associations between continuous variables were examined using Pearson correlation coefficients. Chi-square tests were used to compare BMI classification between WHO and CDC criteria. The significance level was set at *p* < 0.05. Analyses were performed using IBM SPSS Statistics, version 29.

## 3. Results

### 3.1. Participants’ Characteristics

Table 1 presents the demographic and psychological characteristics of the 103 participating parent–child dyads. Most parents were women (77.7%), married (88.3%), and residents of urban areas (67.0%). Regarding parental weight status, 5.8% of parents were underweight, 40.8% had normal weight, while 35.9% were overweight and 17.5% were classified as obese. The educational level was relatively high, with more than 70% of parents having completed higher or postgraduate education.

Among the children, 55.3% were girls and 44.7% boys. According to the World Health Organization (WHO) growth standards [73], 4.9% of children were classified as underweight, 48.5% had normal weight, 17.5% were overweight, 10.7% were obese, and 18.4% were classified as severely obese. Similar distributions were observed using the Centers for Disease Control and Prevention (CDC) criteria. Regarding parental psychological status assessed with the Depression Anxiety Stress Scale-21 (DASS-21), the majority of parents reported scores within the normal range for stress (68.9%), anxiety (72.8%), and depression (75.7%). The prevalence of severe or very severe symptomatology was low across all three subscales, remaining below 8% for each domain.

### 3.2. Correlations Between Parental Factors and Child BMI

Table 2 summarizes the correlations between parental characteristics and children’s BMI (WHO classification). Parental BMI showed a significant positive association with child BMI (r = 0.304, *p* = 0.002), indicating that higher parental weight status was correlated with higher child BMI values.

Child BMI was also positively associated with parental anxiety (r = 0.297, *p* = 0.002) and depression (r = 0.255, *p* = 0.009), suggesting that parental psychological distress may play a role in children’s weight trajectory.

Regarding feeding practices (CFPQ; Comprehensive Feeding Practices Questionnaire), restrictive strategies (r = 0.558, *p* < 0.001), emotional feeding/food as reward (r = 0.466, *p* < 0.001), and child control (r = 0.278, *p* = 0.004) were positively associated with higher child BMI. Conversely, monitoring (r = −0.244, *p* = 0.013), pressure to eat (r = −0.204, *p* = 0.039), and healthy eating guidance (r = −0.318, *p* = 0.001) were negatively associated with child BMI.

No significant correlations were found between child BMI and salivary cortisol indices (AUCg, AUCi).

### 3.3. Associations Between Parental BMI, Psychological Factors, and Feeding Practices

Table 3 displays the relationship between parental BMI, psychological status, and feeding practices. Higher parental BMI was associated with greater perceived stress (r = 0.196, *p* = 0.048), anxiety (r = 0.352, *p* < 0.001), depression (r = 0.262, *p* = 0.008), and more restrictive feeding (r = 0.310, *p* = 0.001).

Parental stress (DASS-21) was positively correlated with child control feeding practices (r = 0.218, *p* = 0.027). Parental anxiety was associated with both emotional feeding (r = 0.307, *p* = 0.002) and restriction (r = 0.218, *p* = 0.027). Depression correlated negatively with monitoring (r = −0.215, *p* = 0.029) and healthy eating guidance (r = −0.331, *p* = 0.001) and positively with emotional feeding (r = 0.199, *p* = 0.044).

### 3.4. Differences by Parental Education Level

As shown in Table 4, parental education was significantly related to parental BMI (*p* = 0.001), child BMI (*p* = 0.002), and restrictive feeding practices (*p* = 0.001). Parents with lower educational attainment (high school graduates, graduates from technical schools) had higher BMI (*p* = 0.001), children with higher BMI (*p* = 0.002), and used more restrictive feeding practices (*p* = 0.001) compared with parents holding postgraduate or doctoral degrees.

### 3.5. Differences by Parental Gender

Table 5 presents comparisons between mothers and fathers. Mann–Whitney U tests were used due to non-normal variable distributions. Fathers had significantly higher BMIs than mothers (*p* = 0.006). Mothers reported significantly greater monitoring of their child’s eating (*p* = 0.020). No significant gender differences were observed for stress, anxiety, depression, or most feeding practice subscales.

### 3.6. Differences by Child Gender

According to Table 6, parents of boys had significantly higher BMIs than parents of girls (*p* = 0.006). Parents of girls demonstrated higher monitoring of their children’s dietary behaviors (*p* = 0.020). All other variables, including child BMI, parental stress, anxiety, and depression, did not significantly differ between boys and girls.

### 3.7. Comparison Between WHO and CDC BMI Classification

Table 7 compares BMI categorization based on WHO versus CDC criteria for 94 children. Of the initial 103 participants, 94 were included because the “underweight” category contained a limited number of cases and was excluded from statistical analysis. WHO identified a slightly higher proportion of overweight and obese children compared with CDC charts. The analysis showed statistically significant differences between the two classification systems (χ^2^ (4) = 159.704, *p* < 0.001), indicating that BMI categorization varies considerably depending on the reference system used.

## 4. Discussion

This study examined the associations between parental psychological functioning, feeding practices, sociodemographic characteristics, and children’s BMI in a Greek sample of 103 parent–child pairs. Moreover, a pilot study that included 13 parents, investigating the use of salivary cortisol as a biological stress marker, was also conducted in a small number of participants. Overall, the findings support the multifactorial nature of childhood obesity and highlight the central role of the family environment, particularly parental mental health and feeding practices, in shaping children’s weight outcomes [61,62,63].

Consistent with the extensive international evidence, parental BMI was strongly associated with child BMI, reinforcing the well-established intergenerational transmission of obesity risk. This link reflects both shared genetic predispositions and shared behavioral and environmental factors within families [29,53,54]. Notably, parental anxiety and depression were also positively related to child BMI. These results suggest that beyond behavioral modeling, the emotional milieu of the household may influence children’s eating patterns and weight status. Previous studies have reported similar associations, indicating that psychological distress can disrupt the parental capacity for consistent and responsive feeding practices and may contribute to unstructured food environments [1,11,12].

Feeding practices demonstrated clear and meaningful associations with children’s BMI. Restrictive feeding, emotional feeding, and greater child control were positively associated with higher child BMI, whereas monitoring and healthy eating guidance were inversely associated. These findings align with previous research showing that restrictive and emotionally driven strategies may undermine children’s ability to self-regulate food intake, increase the desirability of high-fat, high-calorie foods, or encourage maladaptive emotional eating behaviors [18,19,52]. In contrast, responsive practices—such as encouraging healthy eating and monitoring unhealthy food intake—have been linked to healthier dietary patterns and lower obesity risk [15,22,47]. Notably, the observed patterns also mirror cultural dynamics in Mediterranean settings, where food is often intertwined with emotional expression, reward, and parental care [53,67,68].

Educational level emerged as a strong determinant of parental and child weight status as well as feeding practices. Parents with lower educational attainment exhibited higher BMIs, had children with higher BMIs, and used more restrictive feeding practices. These gradients have already been documented in numerous European cohorts and reflect broader socioeconomic inequalities regarding access to health information, nutrition literacy, and lifestyle opportunities [56,57,58]. In Greece, where disparities in health literacy remain prominent, these results underscore the relevance of social determinants in shaping obesity risk [7,59,60].

The comparison between the WHO and CDC classification systems revealed that WHO charts identified a greater proportion of children as overweight or obese. This is consistent with the existing literature reporting the higher sensitivity of WHO references, which are based on optimal growth standards rather than population-based norms [53,54]. For clinical and public health practices, this finding highlights the importance of selecting a classification system aligned with the aims of screening and early identification of at-risk pediatric populations.

No significant associations were detected between salivary cortisol indices and psychological or behavioral variables. This is likely attributable to the limited number of samples obtained during the pilot phase, which severely constrained the statistical power. However, the successful implementation of the saliva collection protocol supports the feasibility of this research approach in future obesity studies. Incorporating biological stress markers may be particularly valuable in stress-related obesity research, providing a measurable biological marker to assess the impact of stress on human behavior and its consequences [69,70].

Overall, the findings point to the importance of addressing parental mental health in the context of childhood obesity prevention frameworks. Interventions that support parents in managing their own stress, enhancing emotional wellbeing, and adopting responsive feeding practices may yield substantial benefits for children’s weight trajectories [11,15,47]. Health promotion programs in Greece should consider integrating parental psychosocial support with traditional nutritional guidance, particularly among families with lower educational attainment [56,57,58]. Future research should expand biomarker assessment, incorporate qualitative approaches to capture cultural nuances in feeding practices, and adopt longitudinal designs to disentangle causal mechanisms. By capturing the interplay between psychological, behavioral, and social determinants, this study contributes to a more comprehensive understanding of childhood obesity within the Greek context.

## 5. Strengths and Limitations

### 5.1. Strengths

This study simultaneously examined parental mental health, feeding practices, and sociodemographic factors in relation to childhood BMI, offering a comprehensive biopsychosocial perspective rarely addressed in Greek populations. The use of validated Greek versions of all psychometric tools (PSS-14, DASS-21, CFPQ), standardized anthropometric methods, and WHO/CDC growth references strengthens the robustness of the findings. An additional strength is the pilot inclusion of salivary cortisol, which, although exploratory, demonstrates the feasibility of integrating biological stress markers in family-based obesity research.

### 5.2. Limitations

The cross-sectional design limits the assessment of causal inferences and the sample—although diverse—was not nationally representative. Self-reported psychological and behavioral measures may be influenced by reporting bias. The relatively small biomarker subsample in the pilot study assessing salivary cortisol as a stress biomarker limited the extraction of meaningful results. Finally, parental measurements collected at home for part of the sample may have introduced measurement variability.

In addition, the analyses were restricted to bivariate associations. Although multivariable models could further clarify independent predictors of child BMI, the present study was designed as an exploratory investigation focusing on the initial relationships between parental psychological factors, feeding practices, and child weight status. Future studies with larger samples and longitudinal designs should incorporate multivariable analytical approaches.

## 6. Conclusions

This study highlights the multifactorial nature of childhood obesity, demonstrating that parental mental health, educational level, and feeding practices jointly influence children’s BMI. Higher parental anxiety, depression, and restrictive or emotion-driven feeding behaviors were associated with increased child BMI, whereas monitoring and health-oriented guidance appeared protective. These findings underscore the need for family-centered prevention strategies that support parental psychological wellbeing and promote responsive, non-restrictive feeding approaches. Although the exploratory salivary cortisol sub-study did not yield significant associations, its feasibility suggests potential value for future biomarker-driven research. Further longitudinal and large-scale studies are required to clarify the causal pathways and inform more targeted public health interventions in Greece and comparable settings.

## Figures and Tables

**Table 1:** Demographic characteristics and psychological status of parents (*n* = 103).

Variable	Category	*n* (%)
Parental sex	Male	23 (22.3)
	Female	80 (77.7)
Parental BMI category	Underweight (<18.5)	6 (5.8)
	Normal weight (18.5–24.9)	42 (40.8)
	Overweight (25.0–29.9)	37 (35.9)
	Obese (≥30.0)	18 (17.5)
Residence	Urban area (Athens)	69 (67.0)
	Non-urban area (Corinthia)	34 (33.0)
Marital status	Single/divorced/widowed	12 (11.7)
	Married	91 (88.3)
Educational level	High school or lower	13 (12.6)
	Vocational/technical training	16 (15.5)
	University degree	34 (33.0)
	Master’s degree	30 (29.1)
	Doctoral degree	10 (9.7)
Occupation	Other occupation	17 (16.5)
	Public sector employee	26 (25.2)
	Private sector employee	41 (39.8)
	Self-employed	19 (18.4)
Child sex	Boy	46 (44.7)
	Girl	57 (55.3)
Child BMI (WHO)	Underweight (−3SD ≤ Z < −2SD)	5 (4.9)
	Normal weight (−1SD ≤ Z ≤ +1SD)	50 (48.5)
	Overweight (+1SD < Z < +2SD)	18 (17.5)
	Obese (+2SD ≤ Z ≤ +3SD)	11 (10.7)
	Severely obese (Z > +3SD)	19 (18.4)
Child BMI (CDC)	Underweight	9 (8.7)
	Normal weight	49 (47.6)
	Overweight	16 (15.5)
	Obese	14 (13.6)
	Severely obese	15 (14.6)
Parental stress (DASS-21 Stress subscale)	Normal (0–14)	71 (68.9)
	Mild (15–18)	12 (11.7)
	Moderate (19–25)	12 (11.7)
	Severe (26–33)	6 (5.8)
	Extreme (34+)	2 (1.9)
Parental anxiety (DASS-21 Anxiety subscale)	Normal (0–7)	75 (72.8)
	Mild (8–9)	6 (5.8)
	Moderate (10–14)	8 (7.8)
	Severe (15–19)	6 (5.8)
	Extreme (20+)	8 (7.8)
Parental depression (DASS-21 Depression subscale)	Normal (0–9)	78 (75.7)
	Mild (10–13)	10 (9.7)
	Moderate (14–20)	10 (9.7)
	Severe (21–27)	3 (2.9)
	Extreme (28+)	2 (1.9)

Note: BMI = Body Mass Index; WHO = World Health Organization; CDC = Centers for Disease Control and Prevention; DASS-21 = Depression Anxiety Stress Scale-21.

**Table 2:** Correlations between parental variables and child Body Mass Index (BMI) based on World Health Organization (WHO) standards (*n* = 103).

Variable	r	*p*
Parental BMI	0.304	0.002 **
Perceived parental stress (PSS-14)	0.076	0.444
Parental stress (DASS-21 Stress subscale)	0.046	0.646
Parental anxiety (DASS-21 Anxiety subscale)	0.297	0.002 **
Parental depression (DASS-21 Depression subscale)	0.255	0.009 **
Monitoring (CFPQ subscale)	−0.244	0.013 *
Child control (CFPQ subscale)	0.278	0.004 **
Emotional feeding/food as reward (CFPQ subscale)	0.466	0.000 **
Pressure to eat (CFPQ subscale)	−0.204	0.039 *
Restriction (CFPQ subscale)	0.558	0.000 **
Healthy eating guidance (CFPQ subscale)	−0.318	0.001 **
Cortisol AUCg (salivary)	0.168	0.584
Cortisol AUCi (salivary)	−0.163	0.594

Note: * *p* < 0.05; ** *p* < 0.01. Pearson correlation coefficients were used. CFPQ = Comprehensive Feeding Practices Questionnaire.

**Table 3:** Correlations between parental psychological factors and child feeding practices (*n* = 103).

Variable 1	Variable 2	r	*p*
Parental BMI	Perceived parental stress (PSS-14)	0.196	0.048 *
Parental BMI	Parental anxiety (DASS-21 Anxiety subscale)	0.352	0.000 **
Parental BMI	Parental depression (DASS-21 Depression subscale)	0.262	0.008 **
Parental BMI	Restriction (CFPQ)	0.310	0.001 **
Parental stress (DASS-21 Stress subscale)	Child control (CFPQ)	0.218	0.027 *
Parental anxiety (DASS-21 Anxiety subscale)	Emotional feeding/food as reward (CFPQ)	0.307	0.002 **
Parental anxiety (DASS-21 Anxiety subscale)	Restriction (CFPQ)	0.218	0.027 *
Parental depression (DASS-21 Depression subscale)	Monitoring (CFPQ)	−0.215	0.029 *
Parental depression (DASS-21 Depression subscale)	Emotional feeding/Food as reward (CFPQ)	0.199	0.044 *
Parental depression (DASS-21 Depression subscale)	Healthy eating guidance (CFPQ)	−0.331	0.001 **

Note: * *p* < 0.05; ** *p* < 0.01. Pearson correlation coefficients were used. CFPQ = Comprehensive Feeding Practices Questionnaire.

**Table 4:** Differences in parental education level across parental and child outcomes (*n* = 103).

Education Level	Parental BMI (Mean ± SD)	Child BMI WHO (Mean ± SD)	Food Restriction (CFPQ) (Mean ± SD)	Perceived Parental Stress (PSS-14) (Mean ± SD)
High school or lower (*n* = 13)	29.04 ± 6.72 †,^	19.19 ± 4.41 †,^	31.08 ± 5.77 †,^	25.62 ± 5.68
Vocational/technical training (*n* = 16)	28.94 ± 6.77 #,<	22.83 ± 6.30 #,<	34.94 ± 8.58 #,<	28.75 ± 8.61
University degree—TEI/AEI (*n* = 34)	25.53 ± 4.80	20.20 ± 5.52	28.53 ± 9.76	22.65 ± 8.47
Master’s degree (*n* = 30)	24.52 ± 5.10 ^,<	16.99 ± 2.92 ^,<	25.13 ± 7.58 ^,<	22.30 ± 5.96
Doctoral degree (*n* = 10)	21.91 ± 4.25 †,#	16.42 ± 4.14 †,#	20.30 ± 7.15 †,#	22.20 ± 8.55
*p*-value (Significance)	0.001 **	0.002 **	0.001 **	0.05 *

Note: * *p* < 0.05; ** *p* < 0.01. One-way ANOVA and Kruskal–Wallis with post hoc pairwise comparisons were conducted to assess differences across education groups. Symbols denote statistically significant pairwise differences: † significantly different from High school or lower, # significantly different from IEK, ^ significantly different from University degree, < significantly different from Doctoral degree.

**Table 5:** Differences in parental variables according to parental sex (*n* = 103).

Variable	Men (Median, IQR)	Women (Median, IQR)	*p* (Two-Tailed)
Parental BMI	28 (4)	25 (4)	0.006 *
Child BMI (WHO)	49 (8)	53 (6)	0.587
Perceived parental stress (PSS-14)	24 (8)	24 (6)	0.651
Parental stress (DASS-21 Stress subscale)	50 (8)	53 (7)	0.721
Parental anxiety (DASS-21 Anxiety subscale)	48 (8)	53 (8)	0.438
Parental depression (DASS-21 Depression subscale)	48 (7)	53 (6)	0.511
Monitoring (CFPQ)	15 (3)	17 (4)	0.020 *
Child control (CFPQ)	49 (6)	53 (6)	0.535
Emotional feeding/food as reward (CFPQ)	57 (7)	51 (7)	0.402
Pressure to eat (CFPQ)	62 (7)	49 (8)	0.059
Healthy eating guidance (CFPQ)	44 (8)	54 (8)	0.155

Note: * *p* < 0.05. Differences were assessed using the independent samples *t*-test and Mann–Whitney U test (two-tailed). CFPQ = Comprehensive Feeding Practices Questionnaire; BMI = Body Mass Index.

**Table 6:** Comparison of boys and girls on key variables (*n* = 103).

Variable	Boys (Median, IQR)	Girls (Median, IQR)	*p*
Child BMI (WHO)	16.2 (2.5)	15.7 (2.6)	0.152
Parental BMI	28 (4.7)	25 (6.7)	0.006 *
Perceived parental stress (PSS-14)	24 (12)	24 (12)	0.651
Parental stress (DASS-21 Stress subscale)	50 (10)	53 (8)	0.721
Parental anxiety (DASS-21 Anxiety subscale)	48 (11)	53 (9)	0.438
Parental depression (DASS-21 Depression subscale)	48 (9)	53 (8)	0.511
Monitoring (CFPQ)	15 (3)	17 (3)	0.020 *
Child control (CFPQ)	49 (7)	53 (6)	0.535
Emotional feeding/food as reward (CFPQ)	57 (10)	51 (7)	0.402
Pressure to eat (CFPQ)	62 (7)	49 (7)	0.059
Healthy eating guidance (CFPQ)	44 (7)	54 (7)	0.155

Note: * *p* < 0.05. Comparisons were performed using the independent samples *t*-tests and Mann–Whitney U test (two-tailed). CFPQ = Comprehensive Feeding Practices Questionnaire; BMI = Body Mass Index.

**Table 7:** Comparison of child BMI classification based on WHO and CDC standards.

BMI Category	WHO *n* (%)	CDC *n* (%)
Normal weight	46 (48.9)	49 (52.1)
Overweight	18 (19.1)	16 (17.0)
Obese	30 (31.9)	29 (30.9)
Total	94 (100)	94 (100)

Note: Data are presented as absolute numbers (*n*) and percentages (%). BMI categories were defined using World Health Organization (WHO) and Centers for Disease Control and Prevention (CDC) growth standards.

## Data Availability

The data supporting the findings of this study are not publicly available due to ethical and privacy restrictions involving minors. De-identified datasets may be provided by the corresponding author upon reasonable request and subject to approval by the institutional ethics committee.
